# Prevalence of *Clostridium difficile* Infection in the Hematopoietic Transplantation Setting: Update of Systematic Review and Meta-Analysis

**DOI:** 10.3389/fcimb.2022.801475

**Published:** 2022-02-21

**Authors:** Ying Luo, Sumei Zhang, Hua Shang, Weitong Cui, Qinglu Wang, Bin Zhu

**Affiliations:** ^1^ Department of Clinical Laboratory, Zibo Central Hospital, Zibo, China; ^2^ Department of Respiratory Medicine, Zibo Central Hospital, Zibo, China; ^3^ Department of Gastroenterology, Zibo Central Hospital, Zibo, China; ^4^ Key Laboratory of Biomedical Engineering & Technology of Shandong High School, Qilu Medical University, Zibo, China; ^5^ College of Sport and Health, Shandong Sport University, Jinan, China

**Keywords:** *Clostridium difficile* infection, hematopoietic stem cell transplantation, meta-analysis, Asia, detection methods, allogeneic transplantation patients

## Abstract

Hematopoietic stem cell transplant (HSCT) recipients are vulnerable to *Clostridium difficile* infection (CDI) due to risk factors such as immunosuppression, antimicrobial use, and frequent hospitalization. We systematically searched PubMed and Embase to screen relevant studies from April 2014 to November 2021. A meta-analysis was performed to identify the association between CDI and hematopoietic transplantation based on the standard mean difference and 95% confidence intervals (CIs). Among the 431 retrieved citations, we obtained 43 eligible articles, which included 15,911 HSCT patients at risk. The overall estimated prevalence of CDI was 13.2%. The prevalence of CDI among the 10,685 allogeneic transplantation patients (15.3%) was significantly higher than that among the 3,840 autologous HSCT recipients (9.2%). Different incidence rates of CDI diagnosis over the last 7 years were found worldwide, of which North America (14.1%) was significantly higher than Europe (10.7%) but not significantly different from the prevalence among Asia (11.6%). Notably, we found that the estimated prevalence of CDI diagnosed by polymerase chain reaction (PCR) (17.7%) was significantly higher than that diagnosed by enzyme immunoassay (11.5%), indicating a significant discrepancy in the incidence rate of CDI owing to differences in the sensibility and specificity of the detection methods. Recurrence of CDI was found in approximately 15% of the initial patients with CDI. Furthermore, 20.3% of CDI cases were severe. CDI was found to be a common complication among HSCT recipients, displaying an evident increase in the morbidity of infection.

## Introduction

*Clostridium difficile* infections (CDI) remain the leading cause of infectious diarrhea among hospitalized patients across the world. The rates of CDI in industrialized countries have increased with the emergence of the NAP1/RT027 strain in 2002, which is responsible for the outbreaks of severe diseases in North America and Europe ( (Loo et al., 2005; Kuijper et al., 2006). Patients with hematologic malignancies—particularly those who undergo hematopoietic stem cell transplants (HSCT)—are at risk of developing CDI because of prolonged hospital stay, exposure to broad-spectrum antibiotics, and compromise of the gastrointestinal mucosal barrier ( (Alonso et al., 2013; Shah et al., 2017).

Given a set of important factors, such as the transplant population, follow-up period, and testing method, the incidence of confirmed CDI among autologous HSCT (auto-HSCT) recipients varies from 5% to 24% (Bruminhent et al., 2014; Pilcante et al., 2015), whereas the incidence among allogeneic HSCT (allo-HSCT) recipients varies from 9% to 34% (Lavallee et al., 2017; Dubberke et al., 2017). An earlier systematic review of published literature until 2014 showed that the pooled prevalence of CDI among 12,025 HSCT patients was 7.9%, and an increasing trend of CDI diagnosis was also found worldwide and across studies conducted in North America over the last 34 years (Zacharioudakis et al., 2014).

Recently, with the widely implemented antibiotic prophylaxis and progress in the diagnostic strategy of CDI, it is unknown how CDI trends change in HSCT recipients during the peri-transplantation and late post-transplantation periods. Therefore, this study evaluated and updated the epidemiology of CDI in the hematopoietic transplantation setting from April 2014 to November 2021.

## Methods

All procedures used in this meta-analysis were consistent with the guidelines of the Meta-analysis of Observational Studies in Epidemiology ( (Stroup et al., 2000) and the Preferred Reporting Items for Systematic Reviews and Meta-analyses (PRISMA) statement.

### Literature Search

We searched PubMed and Embase (April 1, 2014, to November 30, 2021) medical databases to identify publications reporting the prevalence of CDI among patients who received hematopoietic stem cell transplantation. The concise search term was transplant * AND [clostrid * OR difficile OR infect * OR diarrhea OR (*clostridium difficile*) OR (*pseudomembranous colitis*)] AND ([stem cell] OR marrow OR chord OR autologous OR allogeneic) referring to previous systematic reviews (Zacharioudakis et al., 2014). We also manually searched the bibliographies of relevant papers to retrieve additional studies. Articles that were considered eligible following title and abstract reading were assessed in full text.

### Selection Criteria

Studies were considered eligible if they reported the prevalence of CDI among HSCT patients during their hospitalization after stem cell transplantation. A restriction for English literature was imposed.

### Outcomes of Interest

The prevalence of CDI among HSCT patients was the primary outcome of interest in this meta-analysis. CDI was defined as the presence of symptoms (usually diarrhea), and either a stool test positive for *C. difficile* toxins or the presence of toxigenic *C. difficile*, or colonoscopic, or histopathologic findings demonstrating pseudomembranous colitis (McDonald et al., 2018). The prevalence was calculated as the proportion of patients diagnosed with CDI among HSCT recipients. The subgroup analyses included the geographical region, study population, year of study implementation, transplantation type (i.e., autologous or allogeneic), study design, duration of follow-up, and detection methods used in the lab. The recurrence rate of CDI in infected patients was the secondary outcome of interest. Recurrent CDI was defined as a complete elimination of CDI and other symptoms with appropriate therapy, followed by the reappearance of diarrhea and positive result of toxigenic *C. difficile* after the cessation of treatment.

The peri-transplantation period for HSCT patients was divided into four periods: pre-transplantation (pre-T, hospitalization before transplantation), pre-engraftment (pre-E, approximately 0 to 30 days after transplantation), post-engraftment (post-E, approximately 30 to 100 days after transplantation), and late post-transplantation (Lpost-T, generally the day after +100 day of transplantation). Furthermore, to understand the effect of follow-up duration on the estimated prevalence of CDI, we distinguished the duration of follow-up as early- (pre-T + pre-E), middle- (pre-T + pre-E + post-E), and long-term (pre-T + pre-E + post-E + Lpost-T).

### Data Extraction

Two reviewers (YL and QW) independently assessed the studies that were considered for inclusion in the meta-analysis. A spreadsheet was used to summarize the relevant information from the figures, tables, and text of the eligible articles. The trial data published in duplicate were included only once, and the maximum relevant information was extracted. Any disagreements or uncertainties regarding data extraction were resolved in consensus with a third reviewer (BZ). The extracted data included the region of source; study period; patient population; HSCT types (autologous or allogeneic); study design (prospective versus retrospective); laboratory detection methods; source of stem cells; duration of follow-up; the total number of patients who underwent HSCT during the study period; the total number of CDI cases among such patients; the number of NAP1/027 strains; the severity of CDI; and the number of recurrent episodes. If CDI recurred more than once, only the data of the first one were used in the analysis and assessed for the incidence. The severity of CDI in each patient was assessed as severe by the following clinical features: evidence of sepsis, gastrointestinal perforation, pseudomembranous colitis, toxic megacolon, ileus, intensive care unit admission, surgery for colitis, or death because of colitis (Kaltsas et al., 2012). Only studies that mentioned the outcome and severity of CDI were coverage initiated in the analysis.

### Quality Assessment

Two reviewers (YL and QW) independently evaluated the methodological quality of the eligible studies using the Newcastle–Ottawa Quality Assessment Scale, which was a “star-based” rating system. The parameters used to assess the quality of each eligible study were as follows: representative of the exposed cohort, ascertainment of exposure, demonstration that the outcome of interest was not present at the start of the study, assessment of outcome, duration of follow-up for outcomes to occur, and adequacy of follow-up cohorts (Zacharioudakis et al., 2014). Two parameters, selection of the non-exposed cohort and comparability between cohorts, were not applicable to our analysis. Therefore, each study could obtain up to six stars. As representative of the study population in the exposed cohort, we considered the occurrence of CDI among all available transplantation patients rather than a specific subpopulation. We assessed the outcome by presenting the symptoms and laboratory diagnosis of CDI. The follow-up time was viewed as adequate for outcomes to occur, if it was at least 100 days or it included the entire period of hospitalization. Studies that received at least 4 stars were considered adequate quality to extract relevant information.

### Data Analysis

A random-effects model, estimating the pooled prevalence and 95% confidence intervals (CIs), was performed in the meta-analysis (DerSimonian and Laird, 1986). The Freeman–Tukey arcsine methodology was used to remove an excessively large weight for studies with extremely low (close to 0) or extremely high (close to 100%) prevalence (Ziakas et al., 2015). Egger’s test was used to assess the publication bias (Egger et al., 1997). Between-study variance τ^2^ estimation was used to assess statistical heterogeneity (Rucker et al., 2008). Subgroup analyses were used to account for possible sources of heterogeneity. Statistical analysis was implemented by R language software and SPSS software (version 18.0, IBM, New York, USA). The statistical significance threshold was set at 0.05.

## Results

**Figure 1:**
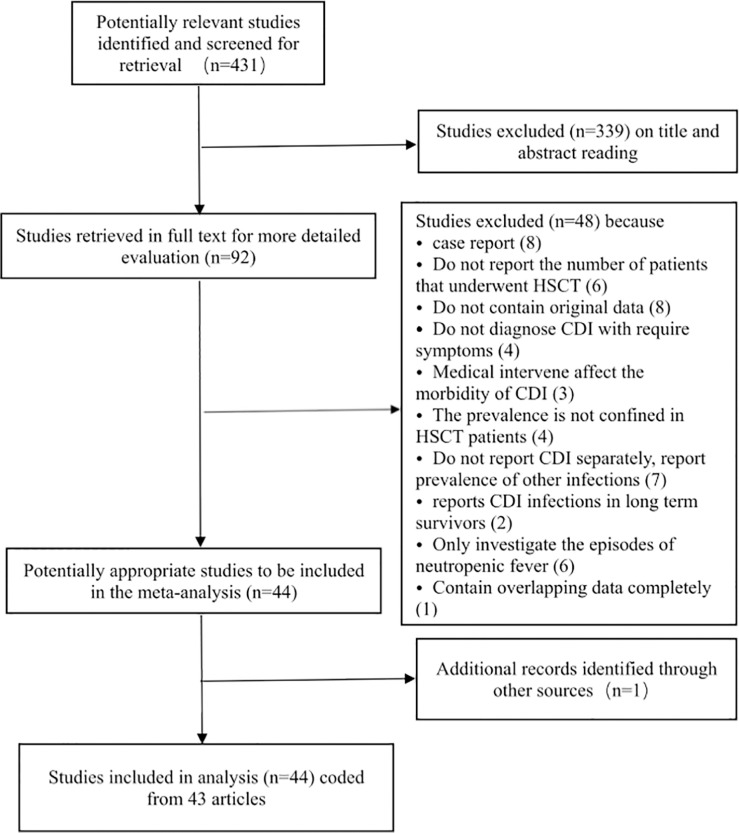
Flowchart of the meta-analysis.

Our search generated 431 publications by accessing the databases between April 1, 2014, and November 30, 2021. After scrutinizing the titles and abstracts of the retrieved articles, 431 studies were excluded from our analysis, and 92 studies were retrieved in full text for more detailed evaluation. Among these, 49 articles were excluded because of the absence of extractable data on the prevalence of CDI among HSCT patients. Of the remaining 43 articles considered suitable for our meta-analysis, two contained partially overlapping data (Schuster et al., 2017; Dubberke et al., 2018), and the maximum available data were extracted from each article. Finally, 44 analyses were included in the final analysis coded from 43 articles (**Table 1**). We presented the details for selecting eligible articles in a flowchart presented in **Figure 1**.

**Table 1:** Characteristics of eligible studies.

Study Citation	Date Source	Study Period	Patient Population	HSCT Types	Study Design	Detection Methods	Follow-up	Source of Stem Cells	N	N-AU	N-AL	n-CDI	n-CDI (AU)	n-CDI (AL)	Recurrence	Quality Score
(Willis et al., 2021)	St. Louis Children’s Hospital, USA	07/2009–02/2018	Ped	AU, AL	Retrospective study	Toxin EIA (2009–2010), GDH EIA, confirmed by a PCR for toxin B (2011–05/2017) and toxin A/B EIA (06/2017–2018)	NR	NR	159	81	78	29	14	15	NR	5
(Jabr et al., 2021)	University of Kansas Medical Center, USA	01/01/2010–12/31/2016	Adult	AL	Retrospective study	Toxin A/B EIA (01/2010–05/2010), and a PCR for toxin B (06/2010–12/2016)	100 days after	PB, BM, UC	656	NR	656	111	NR	111	8	5
(Obeid et al., 2021)	The University of Minnesota, USA	03/2010–06/2015	Adult	AL	Retrospective study	PCR test for toxin B	30 days after	NR	466	NR	466	48	NR	48	12	5
(Weber et al., 2020)	University Hospital Frankfurt, Germany	01/2007–12/2016	Adult	AU, AL	Retrospective study	CD toxin by EIA	30 days before~100 days after	NR	467	191 (lymphoma)	276 (AML)	61	14	47	NR	5
(Majeed et al., 2020)	Banner University Medical Center, USA	11/2013–05/2016	Adult	AU, AL	Retrospective study	GDH, toxin EIA, and a PCR for toxin B (Cepheid), supplement by a cytotoxicity assay	Six months after	NR	180	125	55	17	6	11	2	6
(Austin et al., 2020)	West Virginia University Hospitals, USA	10/2015–06/2017	Adult	AU, AL	Prospective study	GDH and toxin EIA, supplement by a PCR for toxin B	NR	BM, UC	42	16	26	5	NR	NR	NR	6
(Ford et al., 2020)	LDS Hospital, Salt Lake City, USA	06/2015–12/2018	Adult	AU, AL	Retrospective study	GDH and toxin EIA, supplement by a PCR for toxin B (Cepheid)	NR	NR	223	122	101	20	11	9	NR	6
(Rosignoli et al., 2020)	The Transplant Center of Udine, Italy	01/01/2015–12/31/2019	Adult	AU, AL	Retrospective study	GDH and toxin EIA (2015–2017), GDH and toxin EIA supplement by a PCR for toxin B (2018–2019, Cepheid)	100 days after	BM, UC	481	220	261	26	11	15	0	5
(Spruit et al., 2020)	Children’s Hospital of Michigan, USA	01/01/2007–10/31/2017	Ped	AU, AL	Retrospective study	CD toxin by EIA (BD), later by PCR targeting toxin genes (OH)	Whole study period	PB, BM, UC	142	63	79	28	15	13	13 (6/7)	5
(Mardani et al., 2020)	Ayatollah Taleghani University Hospital, Tehran, Iran	05/2017–05/2018	Adult	NR	Prospective study	ELISA A + B kits (Abnova)	NR	BM	43	NR	NR	5	NR	NR	NR	6
(Maakaron et al., 2020)	The Ohio State University, Columbus, USA	07/2015–07/2018	Adult (age, 27–79 years), MM or lymphoma	AU	Retrospective study	NR	NR	BM	514	514	0	51	51	0	NR	5
(Amberge et al., 2020)	University Hospital Carl Gustav Carus, Dresden, Germany	01/01/2004–3/31/2015	Adult, AML, MDS	AL	Retrospective study	CD toxin EIA (Meridian) until 2013, GDH, and toxin EIA (bioMérieux) after 2013	33 months (median)	NR	727	0	727	96	0	96	NR	6
(Rahman et al., 2019)	Cleveland Clinic, OH, USA	2007–2016	Adult (age, 22–76 years), MM	AU	Retrospective cohort study	CD toxin EIA before 2010, and PCR test after 2011	100 days after	PB	413	NR	NR	23	NR	NR	NR	5
(Mullane et al., 2019)	42 Medical centers in North America, USA	NR	Adult (age ≥ 18 years)	AU, AL	Prospective cohort study	Toxin EIA or NAAT (Cepheid Xpert)	60 days after the end of treatment	NR	299	176	123	32	14	18	NR	6
(Ganetsky et al., 2019)	Hospital of the University of Pennsylvania, PA, USA	04/2015–11/2016	Adult	AL	Retrospective cohort study	GDH and toxin EIA, supplement by PCR for toxin genes	30 days before~30 days after	NR	55	0	55	11	0	11	NR	6
(Clemmons et al., 2019)	Augusta University Medical Center, Augusta, USA	2011–2015	Adult (age, 17–75 years)	AU, AL	Retrospective, single-center study	NR	NR	NR	171	115	56	22	14	8	NR	5
(Bhutani et al., 2019)	Columbia University Medical Center, New York, USA	2009–2013	Adult (age, 19–62 years)	AL	Retrospective study	qPCR for CD toxin genes	2.43 years (median)	BM, PB	310	0	310	74	0	74	NR	6
(Salamonowicz et al., 2018)	15 Polish oncological centers, Poland	01/01/2012–12/31/2015	Ped	AU, AL	Retrospective study	EIA, PCR, or culture for toxigenic CD	At least 6 months after	NR	342	75	267	29	5	24	6	6
(Dubberke et al., 2018)	Organ Transplant Infection Project (OTIP), USA	04/2007–03/2010	NR	AL	Prospective multicenter study	EIA for toxins A/B or cytotoxicity assay or antigen detection; PCR or GDH plus toxin EIA	365 days after	NR	385	0	385	120	0	120	NR	6
(Apewokin et al., 2018)	University of Arkansas for Medical Sciences, USA	03/1998–09/2010	MM	AU	Prospective study	CD toxin by EIA (3 samples)	0~21 days after	NR	646	646	0	57	57	0	NR	6
(Schuster et al., 2017)	Organ Transplant Infection Project (OTIP), USA	2006–2011	Adult (age, 18–75 years)	AL	Prospective, multicenter cohort study	NR	30 months after	BM, PB, UC, T-cell depleted	444	0	444	148	0	148	38	5
(Scardina et al., 2017)	Loyola University Medical Center, Maywood, USA	12/01/2009–12/31/2014	NR	AU, AL	Retrospective case–control study	CD toxin EIA (Meridian) until 07/2011, Xpert (Cepheid) after 07/2011	NR	NR	550	NR	NR	44	NR	NR	NR	6
(Lee et al., 2017)	Memorial Sloan Kettering Cancer Center, New York, USA	12/01/2010–11/30/2014	Adult	AL	Prospective study	GeneXpert *C. difficile* toxin assay (Cepheid)	1 year after	NR	234	0	234	53	0	53	15	6
(Lavallee et al., 2017)	University of Montreal, Montreal, Canada	01/01/2002–12/31/2011	Adult	AL	Retrospective case–control study	2002–2005: cytotoxicity assay; 06/2005–01/2010: toxin EIA; 01/2010-2011: GDH and toxin EIA, supplement by cytotoxicity assay	1 year after	BM, PB, UC	760	0	760	65	0	65	6	5
(Dubberke et al., 2017)	Siteman Cancer Center, St. Louis, MO, USA	04/2007– 03/2010	Adult	AL	Prospective cohort study	Remel Xpect *C. difficile* Toxin A/B	30 months after	NR	187	0	187	63	0	63	5	5
(Cannon et al., 2017)	University of Wisconsin School of Medicine and Public Health, Madison, USA	05/12/2015–09/24/2015	NR	NR	Prospective cohort study	Culture and in-house PCR to detect toxin gene	NR	BM	59	NR	NR	5	NR	NR	NR	6
(Aldrete et al., 2017)	Emory University Hospital, Atlanta, USA	11/01/2010–3/31/2013	Adult	AU, AL	Retrospective, case–control study	GeneXpert *C. difficile* toxin assay (Cepheid)	30 days before~90 days after	NR	650	507	143	86	61	25	6	5
(Mani et al., 2016)	Cleveland Clinic, Cleveland, USA	2005–2012	Age range (2–73 years)	AL	Retrospective, single-center study	Toxin EIA before 2010, and PCR test after 2011	6 months before~2 years after	BM, PB, UC	499	0	499	61	0	61	20	5
(Lee et al., 2016)	San Antonio Military Medical Center, Sam Houston, USA	07/2011–04/2014	Adult (age, 19–72 years)	AU, AL	Retrospective, single-center study	Cytotoxin assay or PCR assay	100 days after	NR	77	50	27	8	5	3	NR	5
(Kamboj et al., 2016)	Memorial Sloan Kettering Cancer Center, New York, USA	10/01/2010–12/31/2012	Adult	AL	Prospective study	GeneXpert *C. difficile* toxin assay (Cepheid)	10 days before~40 days after	NR	264	0	264	52	0	52	8	6
(Jain et al., 2016)	Karmanos Cancer Institute and Wayne State University, Detroit, MI, USA	12/01/2010–06/31/2012	NR	NR	Prospective cohort study	Culture and PCR to detect toxin gene	90 days	BM	150	7	143	25	NR	NR	7	6
(Akahoshi et al., 2016)	Saitama Medical Center, Jichi Medical University, Japan	11/2007–05/2014	Adult	AL	Retrospective study	GDH and toxin since 07/2012 (QUIK CHEK COMPLETE, Techlab), and toxin A/B (TOX A/B QUIK CHEK, Techlab)	100 days after	BM, PB, UC	206	0	206	29	0	29	1	5
(Agha et al., 2016)	University of Pittsburgh Medical Center, Pittsburgh, USA	01/2011–12/2014	Adult (age, 22–73 years)	AL	Retrospective cohort study	CD toxin A/B or PCR	28 days after	NR	147	0	147	16	0	16	NR	6
(Pilcante et al., 2015)	Hospital Clínico Universidad Católica, Santiago, Chile	01/2000–06/2013	Adult (age, 17–69 years)	AU, AL	Retrospective study	Toxin EIA from 01/2000 to 02/2012; GeneXpert (Cepheid) at the end of the study	7 days before~365 days after	NR	250	103	147	25	5	20	NR	5
(Gu et al., 2015)	The First Affiliated Hospital of Zhejiang University, Hangzhou, Zhejiang, China	09/01/2009–08/31/2013	Age range (13–77 years)	NR	Retrospective study	Culture and identified by MS (Bruker), then PCR to detect toxin A and B genes	NR	NR	103	NR	NR	14	NR	NR	NR	5
(Boyle et al., 2015)	Fred Hutchinson Cancer Research Center, Seattle, USA	01/01/2008–12/31/2012	Ped, Adult	AL	Prospective study	GDH and toxin EIA (TechLab), supplement by real-time PCR or cytotoxin assay before 2010; GeneXpert (Cepheid) after 2010	56 days before~100 days after	BM, PB, UC	1,182	0	1182	140	0	140	NR	6
(Vehreschild et al., 2014)	University Hospital of Cologne, Cologne, Germany	01/2007–08/2010	Adult	AL	Prospective cohort study	CD toxin A/B EIA (R-Biopharm)	NR	NR	229	0	229	30	0	30	NR	6
(Spadao et al., 2014)	Hospital das Clinicas of University of São Paulo, São Paulo, Brazil	01/2007–06/2011	Age range (12–65 years)	AU, AL	Retrospective study	CD toxin A/B EIA (R-Biopharm)	NR	NR	439	NR	NR	46	15	31	NR	5
(Simojoki et al., 2014)	Helsinki University Central Hospital and University of Helsinki, Helsinki, Finland	01/2007–12/2009	Ped	AL	Retrospective study	CD toxin A/B EIA (bioMerieux)	100 days after	NR	52	0	52	8	0	8	NR	5
(Kinnebrew et al., 2014)	Memorial Sloan-Kettering Cancer Center, New York, USA	09/04/2009–08/04/2011	Adult	AL	Prospective study	Real-time PCR for toxin B gene	15 days before~35 days after	NR	94	0	94	16	0	16		5
		01/01/1999–03/29/2012	NR	AL	Retrospective study	Cytotoxicity assay before 08/29/2008, GDH, and cytotoxicity assay from 08/29/2008 to 09/10/2010, Xpert (Cepheid) after 2010	NR	NR	1,144	0	1144	138	0	138		
(Kamboj et al., 2014)	Memorial Sloan-Kettering Cancer Center, New York, USA	01/01/2005–09/30/2010	Adult, Ped	AL	Retrospective study	Cytotoxin neutralization assay from 01/2005 to 09/2008, GDH, and cytotoxin neutralization assay after 09/2008	10 days before~40 days after	NR	793	0	793	94	0	94		6
(Huang et al., 2014)	University of Michigan Health System (UMHS), Ann Arbor, MI, USA	01/2010–12/2012	NR (mean age, 45 years)	AU, AL	Retrospective case–control study	GDH and toxin EIA, supplement by real-time PCR for toxin genes	7 days before~1 year after	NR	711	381	330	95	35	60	22	5
(Hosokawa et al., 2014)	Toranomon Hospital, Tokyo, Japan	01/2007–12/2008	Adult	AL	Retrospective study	CD toxin A EIA	100 days after	BM, PB, UC	201	0	201	17	0	17	NR	5
(Bruminhent et al., 2014)	Thomas Jefferson University Hospital, Philadelphia, PA, USA	01/2011–12/2012	Adult	AU, AL	Retrospective study	GDH and toxin A/B EIA, supplement by tissue culture cytotoxin assay or molecular assay (Illumigene)	100 days after	BM, PB, UC	150	58	92	37	14	23	3	6

CD, Clostridium difficile; CDI, Clostridium difficile infection; HSCT, hematopoietic stem cell transplantation; Ped, pediatric; MM, multiple myeloma; AML, acute myeloid leukemia; MDS, myelodysplastic syndrome; AU, autologous; AL, allogeneic; EIA, enzyme immunoassay; PCR, polymerase chain reaction; NAAT, nucleic acid amplification tests; NR, unreported; PB, peripheral blood; BM, bone marrow; UC, umbilical cord.

The 44 analyses (coded from 43 articles) included in our analysis were published from April 2014 to November 2021, and data on 15,911 HSCT patients were reported from 1998 to 2019. The characteristics of each study are presented in **Table 1**. In the study containing intervention or prophylaxis that could affect the incidence of CDI among HSCT patients, only the data from the un-intervened cohort were used in the analysis. All studies were considered to possess the adequate quality to be included in the analysis based on the Newcastle–Ottawa Scale (**Supplementary Table 1**).

Among the 44 included analyses, 13 were prospective and 31 were retrospective, and one contained both prospectively and retrospectively collected data. The included studies varied by location, of which 32 were conducted in North America, 6 in Europe, 4 in Asia, and 2 in South America.

**Figure 2:**
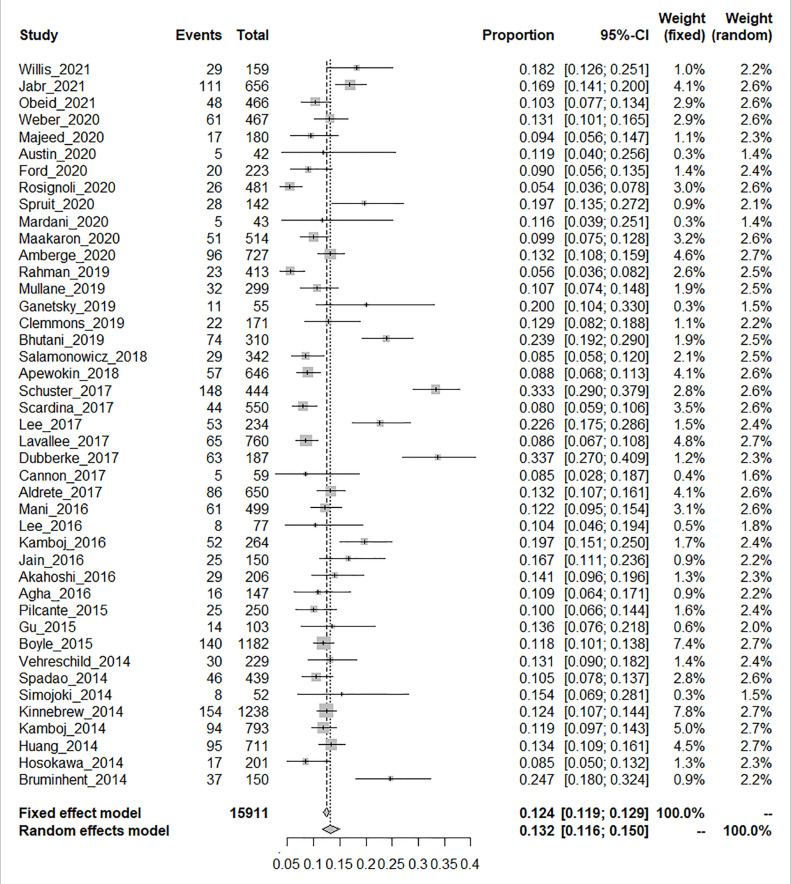
Proportion meta-analysis plot [random-effects model].

The laboratory detection methods of CDI used in each included study are displayed in **Table 1**. The pooled prevalence of CDI among the 15,911 HSCT recipients was 13.2% [95% CI, (11.6% to 15.0%), τ^2^ = 0.0054] according to the random-effects model (**Figure 2**). No evidence of publication bias was found for the overall estimated prevalence according to Egger’s test (bias: 1.654, *p* value = 0.176).

The HSCT patients included in the study were stratified based on age (pediatric or adult) and the type of HSCT (autologous or allogeneic). The included 15,911 HSCT patients included 1,095 pediatric patients extracted from six studies, 10,515 adult patients from 31 studies, and 4,301 patients unidentified by age. No significant diffidence was found in the pooled prevalence of CDI between the pediatric patients [14.8% (95% CI, 10.8% to 19.2%), τ^2^ = 0.0037] and adult patients [13.7% (95% CI, 11.5% to 16.1%), τ^2^ = 0.613] (**Supplementary Figure 1**). Seventeen studies reported relevant data on 3,840 auto-HSCT patients, whereas 34 studies provided extractable data on 10,685 allo-HSCT patients. The prevalence of patients with CDI who underwent allogeneic transplantation was 15.3% [95% CI (13.2% to 17.5%), τ^2^ = 0.0061], which was significantly higher than the corresponding prevalence among auto-HSCT recipients [9.2%, (95% CI, 7.5% to 11.2%), τ^2^ = 0.0026, *p < 0.01*] (**Supplementary Figure 2**).

Among the 43 studies, the estimated prevalence of CDI in North America [14.1% (95% CI, 12.1% to 16.4%), τ^2^ = 0.0063] was higher than the estimated prevalence among European studies [10.7% (95% CI, 7.6% to 14.3%), τ^2^ = 0.0034, *p* = 0.001] but not significantly different from the prevalence among Asian studies [11.6% (95% CI, 8.6% to 14.8%), τ^2^ = 0.0005, *p* = 0.231] (**Supplementary Figure 3**). We also conducted a subgroup analysis on the basis of the population and found that the estimated prevalence of 16 studies with <200 patients [15.8% (95% CI, 12.5% to 19.4%), τ^2^ = 0.0064] was statistically significantly higher than that of 28 studies with ≥200 patients [12.3% (95% CI, 10.5% to 14.2%), τ^2^ = 0.0049, *p < 0.01*] (**Supplementary Figure 4**).

We stratified our data based on the study design (prospective or retrospective) and found that the estimated prevalence of CDI in 13 prospective studies was 16.5% (95% CI, 11.9% to 21.7%), which was significantly higher than that of the 31 retrospective studies [12.0% (95% CI, 10.6% to 13.5%), *p < 0.01*] (**Supplementary Figure 5**). Based on the duration of follow-up, the estimated prevalence of CDI surveyed in the middle term [12.7% (95% CI, 10.5% to 15.2%)] was significantly higher than that in the early term [10.5% (95% CI, 7.9% to 13.4%), *p* = 0.014] and lower than that in the long term [16.5% (95% CI, 12.0% to 21.5%), *p < 0.01*] (**Supplementary Figure 6**).

We also stratified the studies on the detection methods. Forty-one studies expounded on the laboratory detection methods of CDI. The laboratory detection methods of CDI used in each included study are displayed in **Table 1**. Approximately half of the included studies used two or more detection methods to test the *C. difficile* toxin; thereinto, twelve studies altered the detection methods of CDI with time. One or more of the following methods were used in the laboratory detection of CDI: enzyme immunoassay (EIA), tissue culture cytotoxin assay (CC), and polymerase chain reaction (PCR). Ten studies used GDH and/or toxin EIA, supplemented by a PCR for toxin B or culture cytotoxin assay, abbreviated as EIA + PCR/CC. The estimated prevalence of CDI in studies that used EIA + PCR/CC was 14.4% (95% CI, 11.2% to 18%), which was significantly higher than studies that used EIA only [11.5% (95% CI, 9.9% to 13.1%), *p* = 0.02] as well as significantly lower than that in studies that used PCR only [17.7% (95% CI, 13.4% to 22.4%), *p < 0.01*] (**Supplementary Figure 7** and **Table 2**).

**Table 2:** Summary estimates.

CDI	Studies (Articles)	N	Combined Effect (95% CI)	τ^2^	Bias	χ2	p-value
**All studie**s	44 (43)	15,911	13.2% (11.6%–15.0%)	0.0054	1.654		
**Age**						0.256	0.613
Ped	6	1,095	14.8% (10.8%–19.2%)	0.0037	4.536		
Adult	31	10,515	13.7% (11.5%–16.1%)	0.0076	1.919		
**Graft type**						70.990	0.000
Autologous	17	3,840	9.2% (7.5%–11.2%)	0.0026	1.168		
Allogeneic	34 (33)	10,685	15.3% (13.2%–17.5%)	0.0061	1.806		
**Population**						30.709	0.000
≥200 patients	28	14,100	12.3% (10.5%–14.2%)	0.0049	1.546		
<200 patients	16	1,811	15.8% (12.5%–19.4%)	0.0064	-2.203		
**Geographical region**							
North America	32 (31)	12,371	14.1% (12.1%–16.4%)	0.0063	2.352		Ref
Europe	6	2,298	10.7% (7.6%–14.3%)	0.0034	0.762	11.966	0.001
Asia	4	553	11.6% (8.6%–14.8)	0.0005	0.762	1.436	0.231
**Study design**						50.827	0.000
Prospective	13	3,873	16.5% (11.9%–21.7%)	0.0125	1.806		
Retrospective	31	12,038	12.0% (10.6%–13.5%)	0.0029	1.335		
**Duration of follow-up**							
Early term	3	1,314	10.5% (7.9%–13.4%)	0.0010	2.876	6.002	0.014
Middle term	16	6,135	12.7% (10.5%–15.2%)	0.0039	1.409		Ref
Long term	11	4,786	16.5% (12.0%–21.5%)	0.0116	5.737	24.227	0.000
**Detection method**							
EIA	9	3,010	11.5% (9.9%–13.1%)	0.0005	0.713	5.449	0.020
EIA+PCR/CC	10	3,078	14.4% (11.2%–18.0%)	0.0044	1.984		Ref
PCR	10	2,517	17.7% (13.4%–22.4%)	0.0074	2.146	14.991	0.000
**Detection years**						15.531	0.000
Before 2010s	7	3,120	10.1% (8.7%–11.7%)	0.0004	1.393		
After 2010s	21	14,100	12.3% (10.5%–14.2%)	0.0049	0.952		

CDI, Clostridium difficile infection; Ped, pediatric; EIA, enzyme immunoassay; PCR, polymerase chain reaction; CC, culture cytotoxin assay; Ref, reference.

Eighteen studies reported data on recurrence of CDI among 990 infected patients, among which 11 studies included the definition of recurrence. The reported recurrence rate was estimated to be 14.9% [95% CI (9.8% to 20.7%), τ^2^ = 0.0193] (**Supplementary Figure 8**). The individual study data of the first recurrent case are presented in **Table 1**. Further analyses were performed for the estimated prevalence of CDI patients from 1998 to 2010 and from 2011 to 2021; the results showed that the estimated prevalence of CDI in 1998–2010 patients was 10.1% (95% CI, 8.7% to 11.7%), which was significantly lower than that of the 2011–2021 patients [13.0% (95% CI, 10.9% to 15.3%), *p < 0.01*] (**Supplementary Figure 9**).

Finally, seven studies which included the definition of CDI severity, and 11 studies which reported data on the severity of CDI among infected patients, were included in the analysis. Among 524 CDI patients, 107 (20.3%, 107/524) severe cases, 26 (5.0%, 26/524) ICU admissions, 9 (1.7%, 9/524) CDI-related colectomies, 7 (1.3%, 7/524) gastrointestinal perforations, 13 (2.5%, 13/524) pseudomembranous colitis cases, and 13 (2.5%, 13/524) deaths were reported in the remaining 11 studies. Two studies reported high-virulent NAP1/027 strains, in one of which NAP1/027 strains account for 24.5% (23/94), in the other one only 2.7% (1/37) (**Supplementary Table 2**).

## Discussion

CDI has been increasingly discerned among HSCT recipients because of the fragility of the immune system, graft-versus-host disease (GVHD), and antibiotic usage or prophylaxis (Ilett et al., 2019; Rosignoli et al., 2020; Jabr et al., 2021). Along with the growing cognition on CDI for clinical physicians and improving diagnostic capacity of laboratories on CDI, the relevant data on the prevalence of reported CDI have gradually increased in recent years. This study aimed to update the previous analysis on the prevalence of CDI among HSCT patients by Zacharioudakis et al. (Zacharioudakis et al., 2014) and investigate the variation in the estimated prevalence and subgroup analysis of CDI among HSCT recipients reported from April 1, 2014, to November 30, 2021.

In our study, the estimated prevalence of CDI in HSCT patients was 13.2%, which was approximately two times higher than the corresponding morbidity reported in the previous analysis (7.9%) (Zacharioudakis et al., 2014) and approximately 15 times higher than the general hospital population (0.9% reported in 2009) (Lucado et al., 2006). In the analysis by Zacharioudakis, the actual change in *C. difficile* epidemiology was attributed to the emergency of more virulent strains (Zacharioudakis et al., 2014). However, we had different findings in the variability of *C. difficile* epidemiology because of several factors, including spectrum antibiotics, immunosuppression, strain, and the diagnostic sensitivity of CDI (Alonso et al., 2013; Shah et al., 2017). Analysis of our data between 1998–2010 and 2011–2021 also showed a gradual increase in prevalence of CDI among HSCT recipients.

The prevalence of allogeneic transplantation patients was 15.3%, which was significantly higher than autologous graft (9.2%), indicating that the graft type was one of the primary elements to influence the prevalence of CDI among HSCT recipients. The risk factors for CDI in allo-HSCT patients included receipt of chemotherapy before conditioning for HSCT, broad-spectrum antimicrobial use, acute GVHD, and greater immunosuppression caused by allo-HSCT conditioning regimens (Alonso et al., 2012). A greater deviation in the prevalence of CDI compared to the overall estimated prevalence (13.2%) was found for smaller studies (<200 patients, 15.8% vs. ≥200 patients, 12.3%), highlighting that a reasonable and large sample size was necessary for reducing the random error and being representative.

In our analysis, we observed that most of the studies (72.1%, 31/43) were obtained from North America, and the estimated prevalence of CDI among HSCT patients in North America was 14.1%, which was significantly higher than that in Europe (10.7%) but did not reach statistical significance than that in Asia (11.6%). It revealed the regional epidemic characteristics of CDI over the last 7 years. Another national discharge data also indicated that the USA had a 10-fold higher CDI rate than England among overall inpatients (King et al., 2017). The regional difference might be associated with the national infection control policy or epidemic of a hypervirulent strain. Therefore, continuous regional surveillance was necessary to investigate the presumed association between vulnerability and CDI in the different ethnic groups and regions.

In our study, we only included data on the first post-transplant hospitalization, which may have resulted in the higher overall estimated prevalence. Most studies were followed up from pre-transplantation to 100 days post-transplantation, and the estimated prevalence of CDI with the middle term of follow-up was 12.7%, which was significantly higher than the early term (*p* = 0.014) and significantly lower than the long term (*p* < 0.01). However, most cases of CDI among HSCT recipients were diagnosed in the early term of transplantation because of more intense antimicrobial exposure, high immunosuppression, accelerated antimicrobial exposure, and increased transmission in the hospital environment (Schuster et al., 2017). Our study indicated that the risk of CDI among the middle and late periods cannot be ignored.

The diagnosis of CDI is a complicated process, incorporating clinical diagnosis, defined by the presence of symptoms (usually diarrhea), with laboratory diagnosis, assured by either a stool test positive for *C. difficile* toxin or detection of toxigenic *C. difficile* or colonoscopic or histopathologic findings revealing pseudomembranous colitis (McDonald et al., 2018). In our studies, the estimated prevalence of CDI diagnosed by EIA (11.5%) was significantly lower than that diagnosed by EIA+PCR/CC (14.4%, *p* = 0.02), and the CDI diagnosed by EIA+PCR/CC was significantly lower than that diagnosed by PCR (17.7%, *p* < 0.01), indicating that a significant discrepancy in the incidence rate of CDI was observed because of the different sensibility and specificity of the detection methods of CDI. The related laboratory indices of CDI diagnosis detected by EIA were glutamate dehydrogenase (GDH) and *C. difficile* toxin A and/or B (CDAB). One of our previous studies revealed that the sensitivity of the detection method combining GDH and CDAB for the diagnosis of CDI was only 54.2% (39/72), and with further addition of PCR to the scheme, the sensitivity for the diagnosis of CDI could be increased to 100% (Luo et al., 2018). This mate analysis showed that a PCR for CD toxin was the most sensitive detection method for CDI. A conventional PCR for CD toxin needs to be combined with time-consuming and demanding anaerobic culture, increasing the difficulty of its universal use. In recent years, some commercially nucleic acid amplification test (NAAT) products were approved by the FDA, such as the Gene Xpert CD assay (Cepheid, Sunnyvale, USA) directly detecting the *tcdB* gene in feces by RT-PCR, and widely used in the national world. The Gene Xpert was notable because of its high sensitivity (97%) and specificity (95%) in diagnosing toxigenic CDI both rapidly and simply ( (Bai et al., 2017).

The recurrence of CD infection occurred in approximately 15% of the initial patients with CDI, with a large variation from 3% to 46% in our analysis. Antecedent antibiotic usage and neutropenia were considered independent predictors of recurrent CDI (Huang et al., 2014; Mani et al., 2016). Notably, 20.3% of CDI cases were severe. However, because of failing raw data on each risk factor, further statistical statements could not be implemented in our analysis. Infection control measures and regional epidemiology possess a significant role in the prevalence of CDI among individual medical centers, and our pooled estimation does not reduce the need for local centers to understand local prevalence. The meta-analysis showed that fecal microbiota transplantation, as an innovative strategy to reduce CDI occurrence, was recommended in patients with recurrent CDI in whom appropriate antibiotic treatments failed (Pession et al., 2021).

Our study estimated the pooled prevalence of CDI among HSCT recipients to be almost 2-fold higher than that in the previous analysis (Zacharioudakis et al., 2014). The increased prevalence of CDI with the high rate of severe cases highlighted the necessity for prophylactic policies, such as antimicrobial stewardship programs, strict hand hygiene procedures, and environmental decontamination that is specifically aimed at this patient population. Furthermore, future studies were required to recognize immunosuppressive and preventive antimicrobial regimens that were presumedly associated with a lower risk of CDI.

## Data Availability Statement

The original contributions presented in the study are included in the article/**Supplementary Material**. Further inquiries can be directed to the corresponding author.

## Author Contributions

Conceptualization: QW. Data curation: YL, BZ, SZ, and HS. Software: YL and WC. Writing—original draft: YL, BZ, and QW. Writing—review and editing: YL, SZ, and HS. All authors contributed to the article and approved the submitted version.

## Funding

This work was supported by grants from the Natural Scientific Foundation of Shandong Province, China (ZR2018MH038, ZR2019PC053) and the Zibo City Innovation Development Key Project (2018CX04A007).

## Conflict of Interest

The authors declare that the research was conducted in the absence of any commercial or financial relationships that could be construed as a potential conflict of interest.

## Publisher’s Note

All claims expressed in this article are solely those of the authors and do not necessarily represent those of their affiliated organizations, or those of the publisher, the editors and the reviewers. Any product that may be evaluated in this article, or claim that may be made by its manufacturer, is not guaranteed or endorsed by the publisher.

## Supplementary Material

Supplementary Table 1 Newcastle Ottawa Quality assessment of individual studies.

Supplementary Table 2 Recurrence and outcomes of CDI in HSCT recipients.

Supplementary Figure 1 Prevalence of CDI among adult **(A)** and pediatric **(B)** HSCT patients.

Supplementary Figure 2 Prevalence of CDI among allogeneic **(A)** and autologous **(B)** HSCT recipients.

Supplementary Figure 3 Prevalence of CDI among studies in North America **(A)**, Asia **(B)**, and Europe **(C)**.

Supplementary Figure 4 Prevalence of CDI among studies with < 200 patients **(A)** and studies with ≥ 200 patients **(B)**.

Supplementary Figure 5 Prevalence of CDI in prospective studies **(A)** and retrospective studies **(B)**.

Supplementary Figure 6 Prevalence of CDI surveyed in the Early term **(A)**, Middle term **(B)**, and Long term **(C)**.

Supplementary Figure 7 Prevalence of CDI in studies with EIA used only **(A)**, EIA + PCR/CC **(B)**, and PCR used only **(C)**.

Supplementary Figure 8 Prevalence of recurrent CDI in studies.

Supplementary Figure 9 Prevalence analysis of CDI in 1998-2010 **(A)** and 2010-2020 **(B)** year.
