# Supplementary material for: Prevalence of Clostridium difficile Infection in the Hematopoietic Transplantation Setting: Update of Systematic Review and Meta-Analysis
Source: Front Cell Infect Microbiol. 2022 Feb 21;12:801475. doi: 10.3389/fcimb.2022.801475 (PMC8900492; doi:10.3389/fcimb.2022.801475)
Supplement: Supplementary Table 1 — Newcastle Ottawa Quality assessment of individual studies. [file Table_1.docx]

Supplementary Table 1. Newcastle Ottawa Quality assessment of individual studies.

| Author_ year | Selection | | | | Comparability | Outcome | | | Total score |
| --- | --- | --- | --- | --- | --- | --- | --- | --- | --- |
|  | Representativeness of exposed cohort | Selection of the non-exposed cohort | Ascertainment of exposure | Demonstration that outcome of interest was not present at start of study | Comparability of cohorts on the basis of the design or analysis | Assessment of outcome | Follow-up long enough for outcomes to occur | Adequacy of follow-up of cohorts |  |
| Willis_2021 | 1 | 1 | 1 | 1 | 1 |  |  |  | 5 |
| Jabr_2021 | 1 |  | 1 | 1 |  | 1 | 1 |  | 5 |
| Obeid_2021 | 1 | 1 | 1 | 1 |  | 1 |  |  | 5 |
| Weber_2020 | 1 |  | 1 | 1 |  | 1 | 1 |  | 5 |
| Majeed_2020 | 1 |  | 1 | 1 | 1 | 1 | 1 |  | 6 |
| Austin­_2020 | 1 | 1 | 1 | 1 | 1 | 1 |  |  | 6 |
| Ford_2020 | 1 |  | 1 | 1 | 1 | 1 |  | 1 | 6 |
| Rosignoli_2020 | 1 |  | 1 | 1 |  | 1 |  | 1 | 5 |
| Spruit_2020 | 1 |  | 1 | 1 |  | 1 | 1 |  | 5 |
| Mardani_2020 | 1 | 1 | 1 | 1 | 1 |  | 1 |  | 6 |
| Maakaron_2020 | 1 |  | 1 | 1 |  | 1 | 1 |  | 5 |
| Amberge_2020 | 1 |  | 1 | 1 |  | 1 | 1 | 1 | 6 |
| Rahman_2019 | 1 |  | 1 | 1 | 1 |  | 1 |  | 5 |
| Mullane_2019 | 1 |  | 1 | 1 |  | 1 | 1 | 1 | 6 |
| Ganetsky_2019 | 1 |  | 1 | 1 | 1 | 1 | 1 | 1 | 7 |
| Clemmons_2019 | 1 | 1 | 1 | 1 |  | 1 |  |  | 5 |
| Bhutani_2019 | 1 |  | 1 | 1 | 1 | 1 | 1 |  | 6 |
| Salamonowicz_2018 | 1 |  | 1 | 1 |  | 1 | 1 | 1 | 6 |
| Dubberke_2018 | 1 |  | 1 | 1 |  | 1 | 1 | 1 | 6 |
| Apewokin_2018 | 1 | 1 | 1 | 1 |  | 1 |  | 1 | 6 |
| Schuster_2017 | 1 |  | 1 |  | 1 | 1 |  | 1 | 5 |
| Scardina_2017 | 1 | 1 | 1 | 1 |  | 1 | 1 |  | 6 |
| Lee_2017 | 1 |  | 1 | 1 |  | 1 | 1 | 1 | 6 |
| Lavallee_2017 | 1 |  | 1 | 1 |  | 1 | 1 |  | 5 |
| Dubberke_2017 | 1 |  | 1 | 1 |  | 1 | 1 |  | 5 |
| Cannon_2017 | 1 | 1 | 1 | 1 |  | 1 | 1 |  | 6 |
| Aldrete_2017 | 1 |  | 1 | 1 | 1 |  | 1 |  | 5 |
| Mani_2016 | 1 |  | 1 |  |  | 1 | 1 | 1 | 5 |
| Lee_2016 | 1 | 1 | 1 | 1 |  | 1 |  |  | 5 |
| Kamboj_2016 | 1 |  | 1 | 1 | 1 | 1 | 1 |  | 6 |
| Jain_2016 | 1 |  | 1 | 1 |  | 1 | 1 | 1 | 6 |
| Akahoshi_2016 | 1 |  | 1 | 1 |  | 1 | 1 |  | 5 |
| Agha_2016 | 1 | 1 | 1 | 1 |  | 1 | 1 | 1 | 7 |
| Pilcante_2015 | 1 |  | 1 | 1 | 1 | 1 |  |  | 5 |
| Gu_2015 | 1 |  | 1 | 1 |  |  | 1 | 1 | 5 |
| Boyle_2015 | 1 | 1 | 1 | 1 |  | 1 | 1 |  | 6 |
| Vehreschild_2014 | 1 |  | 1 | 1 | 1 | 1 |  | 1 | 6 |
| Spadao_2014 | 1 |  | 1 | 1 |  | 1 | 1 |  | 5 |
| Simojoki_2014 | 1 |  | 1 | 1 |  | 1 | 1 |  | 5 |
| Kinnebrew_2014 | 1 |  | 1 | 1 |  | 1 | 1 |  | 5 |
| Kamboj_2014 | 1 | 1 | 1 | 1 |  | 1 | 1 |  | 6 |
| Huang_2014 | 1 |  | 1 |  |  | 1 | 1 | 1 | 5 |
| Hosokawa_2014 | 1 |  | 1 | 1 |  | 1 | 1 |  | 5 |
| Bruminhent_2014 | 1 | 1 | 1 | 1 |  | 1 | 1 |  | 6 |

The concise search term was transplant * AND (clostrid * OR difficile OR infect * OR diarrhea OR [*clostridium difficile*] OR [*pseudomembranous colitis*]) AND ([stem cell] OR marrow OR chord OR autologous OR allogeneic) refer to the previous systematic reviews(Zacharioudakis, Ziakas, and Mylonakis 2014)
