# Supplementary material for: Prevalence of Clostridium difficile Infection in the Hematopoietic Transplantation Setting: Update of Systematic Review and Meta-Analysis
Source: Front Cell Infect Microbiol. 2022 Feb 21;12:801475. doi: 10.3389/fcimb.2022.801475 (PMC8900492; doi:10.3389/fcimb.2022.801475)
Supplement: Supplementary Table 2 — Recurrence and outcomes of CDI in HSCT recipients. [file Table_2.docx]

Supplementary Table 2: Recurrence and outcomes of CDI in HSCT recipients.

| Study citation | HSCT types | N | n-CDI | Recurrence  (No, of cases) | Recurrence definition | Time of CDI diagnosis | Severity definition | Outcomes  (No, of cases) |
| --- | --- | --- | --- | --- | --- | --- | --- | --- |
| Willis_2021 | AU, AL | 159 | 29 | NR | \ | \ | \ | \ |
| Jabr_2021 | AL | 656 | 111 | 8 | \ | \ | \ |  |
| Obeid_2021 | AL | 466 | 48 | 12 | Recurrent CDI was a new diagnosis of CDI 8 weeks to 6 months from the index CDI event. | \ | \ | \ |
| Weber_2020 | AU, AL | 467 | 61 | NR | \ | medium: 9.5 days (range -12～+95days) after HSCT. | \ | \ |
| Majeed_2020# | AU, AL | 180 | 17 | 2 | \ | \ | The severity of CDI was classified as mild (grade 1 diarrhea/colitis), moderate (grade 2 diarrhea/colitis) and severe (grade ≥3 diarrhea/colitis) based on symptoms noted at 48 hours before or after CDI diagnosis. | No patient suffered from toxic megacolon or ileus, and no patient underwent colectomy. There was no mortality associated with CDI at our center. |
| Austin­_2020 | AU, AL | 42 | 5 | NR | \ | \ | \ | \ |
| Ford_2020 | AU, AL | 223 | 20 | NR | \ | \ | \ | \ |
| Rosignoli_2020 | AU, AL | 481 | 26 | 0 | \ | medium: 12 days (range -5～+100days) after HSCT. | The severity of the gastrointestinal symptoms was graded according to National Cancer Institute Common Terminology Criteria for Adverse Event (CTCAE 3.0). | \ |
| Spruit_2020 | AU, AL | 142 | 28 | 13（6/7） | Recurrence of CDI was defined as a subsequent positive test after resolution of symptoms, completion of a course of antibiotics, and at least 30 days from the previous positive test. | \ | \ | \ |
| Mardani_2020 | \ | 43 | 5 | \ | \ | \ | \ | \ |
| Maakaron_2020 | AU | 514 | 51 | \ | \ | \ | \ | \ |
| Amberge_2020 | AL | 727 | 96 | \ | \ | \ | \ | \ |
| Rahman_2019 | AU | 413 | 23 | \ | \ | \ | \ | \ |
| Mullane_2019 | AU, AL | 299 | 32 | \ | \ | \ | \ | \ |
| Ganetsky_2019 | AL | 55 | 11 | \ | \ | \ | \ | \ |
| Clemmons_2019# | AU, AL | 171 | 22 | \ | \ | \ | \ | ICU admission: 3 |
| Bhutani_2019 | AL | 310 | 74 | \ | \ | \ | \ | \ |
| Salamonowicz_2018# | AU, AL | 342 | 29 | 6 | CDI recurrence was defined as a new episode of clinically and microbiologically documented CDI separated by complete resolution of clinical symptoms of primary infection with at least 4 weeks interval. | medium: 21 days (range 2–180 days) after HSCT. | Severe CDI course was defined by CDI first-line treatment failure which meant lack of improvement in stool consistency after 3 days, the need to add the second drug, new signs of severe colitis, the need of admission to an intensive care unit, and/or death related to CDI | Mortality: 1 |
| Dubberke_2018 | AL | 385 | 120 | \ | \ | The median time from transplant to CDI was 27 days among allogeneic HCT patients | \ | \ |
| Apewokin_2018 | AU | 646 | 57 | \ | \ | \ | \ | \ |
| Schuster_2017# | AL | 444 | 148 | 38 | \ | The median time to CDI was 27 days posttransplant. | \ | ICU admission: 19  Mortality: 12  Colectomy: 1  Gastrointestinal perforation: 2  Pseudomembranous colitis: 2 |
| Scardina_2017# | AU, AL | 550 | 44 | \ | \ | \ | \ | Severe CDI: 16  Colectomy: 2  Gastrointestinal perforation: 1  Pseudomembranous colitis: 3 |
| Lee_2017# | AL | 234 | 53 | 15 | CDI recurrence was defined as symptomatic recurrence ≥14 days after the initial episode. | \ | CDI severity was defined as low (outpatient illness or uncomplicated inpatient management without need for imaging), medium (colitis at imaging), or severe (associated with clinical sepsis) intensive care unit admission, death due to colitis, or pseudomembranous colitis) | Severe:5  Colectomy: 1  Pseudomembranous colitis: 1 |
| Lavallee_2017 | AL | 760 | 65 | 6 | Recurrence was defined as another episode of symptomatic CDI occurring within 8 weeks after completion of treatment. | a median onset of CDI was 45 days after transplantation, | \ | \ |
| Dubberke_2017# | AL | 187 | 63 | 5 | \ | \ | CDI cases were classified by severity (mild, moderate, severe) according to modified Common Terminology Criteria for Adverse Events (CTCAE)criteria | Severe: 17  Colectomy: 2  Gastrointestinal perforation: 2  Pseudomembranous colitis: 4 |
| Cannon_2017 | \ | 59 | 5 | \ | \ | \ | \ | \ |
| Aldrete_2017 | AU, AL | 650 | 86 | 6 | \ | Median day: 5 days | \ | \ |
| Mani_2016 | AL | 499 | 61 | 20 | Recurrent CDI is defined as any CDI that occurs after the first appropriately treated episode with 10–14 days of either oral metronidazole or vancomycin | \ | \ | \ |
| Lee_2016 | AU, AL | 77 | 8 | \ | \ | The median time from transplant to CDI was 5 days (range 2–100) | \ | \ |
| Kamboj_2016# | AL | 264 | 52 | 8 | Recurrence was defined as a CDI episode that occurred 2 weeks after the index event. | \ | \ | ICU admission: 1 |
| Jain_2016# | \ | 150 | 25 | 7 | Recurrent CDI was defined as a new onset of diarrhea and a C. difficile positive stool PCR assay within 90 days of previous CDI | median time: 12 days | \ | Severe cases: 0 |
| Akahoshi_2016 | AL | 206 | 29 | 1 | Recurrence was defined as a new episode of diarrhea and a positive toxin EIA within 365 d after the first episode of CDI. | median time: 7 days | \ | \ |
| Agha_2016 | AL | 147 | 16 | \ | \ | \ | \ | \ |
| Pilcante_2015# | AU, AL | 250 | 25 | \ | \ | median time: 21 days | \ | Severe disease: 2 |
| Gu_2015 | \ | 103 | 14 | \ | \ | \ | \ | \ |
| Boyle_2015 | AL | 1182 | 140 | \ | \ | Ped：51days adult：16 days | \ | \ |
| Vehreschild_2014 | AL | 229 | 30 | \ | \ | \ | \ | \ |
| Spadao_2014# | AU, AL | 439 | 46 | \ | \ | \ | Severe cases: Patients presented with one or more of the following variables during the treatment of diarrhea: hypotension; shock, renal insufficiency (50% decreases in creatinine clearance), toxic megacolon; colectomy and death within up to 30 days of onset of clinical symptoms. | ICU admission: 3  Colectomy: 2  Gastrointestinal perforation: 2  Pseudomembranous colitis: 3 |
| Simojoki_2014 | AL | 52 | 8 | \ | \ | \ | \ | \ |
| Kinnebrew_2014 | AL | 94 | 16 | \ | \ | \ | \ | \ |
|  | AL | 1144 | 138 | \ | \ | \ | \ | \ |
| Kamboj_2014 | AL | 793 | 94 | \ | \ | \ | \ | NAP1/027: 23 (24%.) |
| Huang_2014 | AU, AL | 711 | 95 | 22 | Recurrence was defined as a repeat positive CDI occurring within 6 months after the end of treatment for the index case (first episode of CDI after transplantation). | \ | Severe CDI was defined as an episode of CDI resulting in need for intensive care unit admission, interventional surgery, or death within 30 days of CDI diagnosis | \ |
| Hosokawa_2014 | AL | 201 | 17 | 0 | Recurrence was defined as a new episode of diarrhea with positive toxin assay results within 8 weeks after improvement of the first properly treated episode. | Median time: 22 days | \ | \ |
| Bruminhent_2014 | AU, AL | 150 | 37 | 3 | \ | 3.5 days | \ | NAP1/027: 1 |

Abbreviations: CD: *Clostridium difficile*; CDI: *Clostridium difficile* infection; HSCT: hematopoietic stem cell transplantation; AU: autologous; AL: allogeneic.

The concise search term was transplant * AND (clostrid * OR difficile OR infect * OR diarrhea OR [*clostridium difficile*] OR [*pseudomembranous colitis*]) AND ([stem cell] OR marrow OR chord OR autologous OR allogeneic) refer to the previous systematic reviews(Zacharioudakis, Ziakas, and Mylonakis 2014).

Note: #studies included in the analysis of severity of CDI.
