# Supplementary figures and images for: Prevalence of Clostridium difficile Infection in the Hematopoietic Transplantation Setting: Update of Systematic Review and Meta-Analysis
Source: Front Cell Infect Microbiol. 2022 Feb 21;12:801475. doi: 10.3389/fcimb.2022.801475 (PMC8900492; doi:10.3389/fcimb.2022.801475)

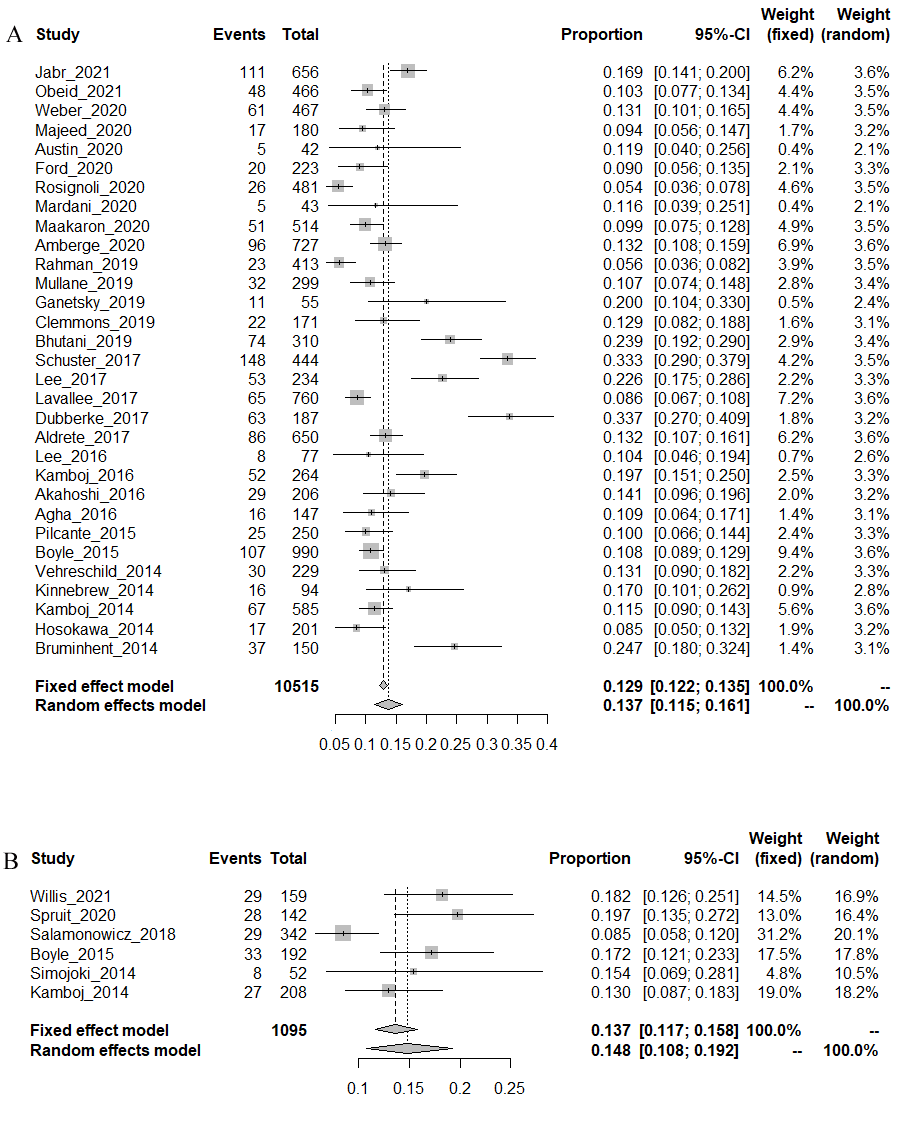

Supplement: Supplementary Figure 1 — Prevalence of CDI among adult (A) and pediatric (B) HSCT patients. [file Image_1.tif]

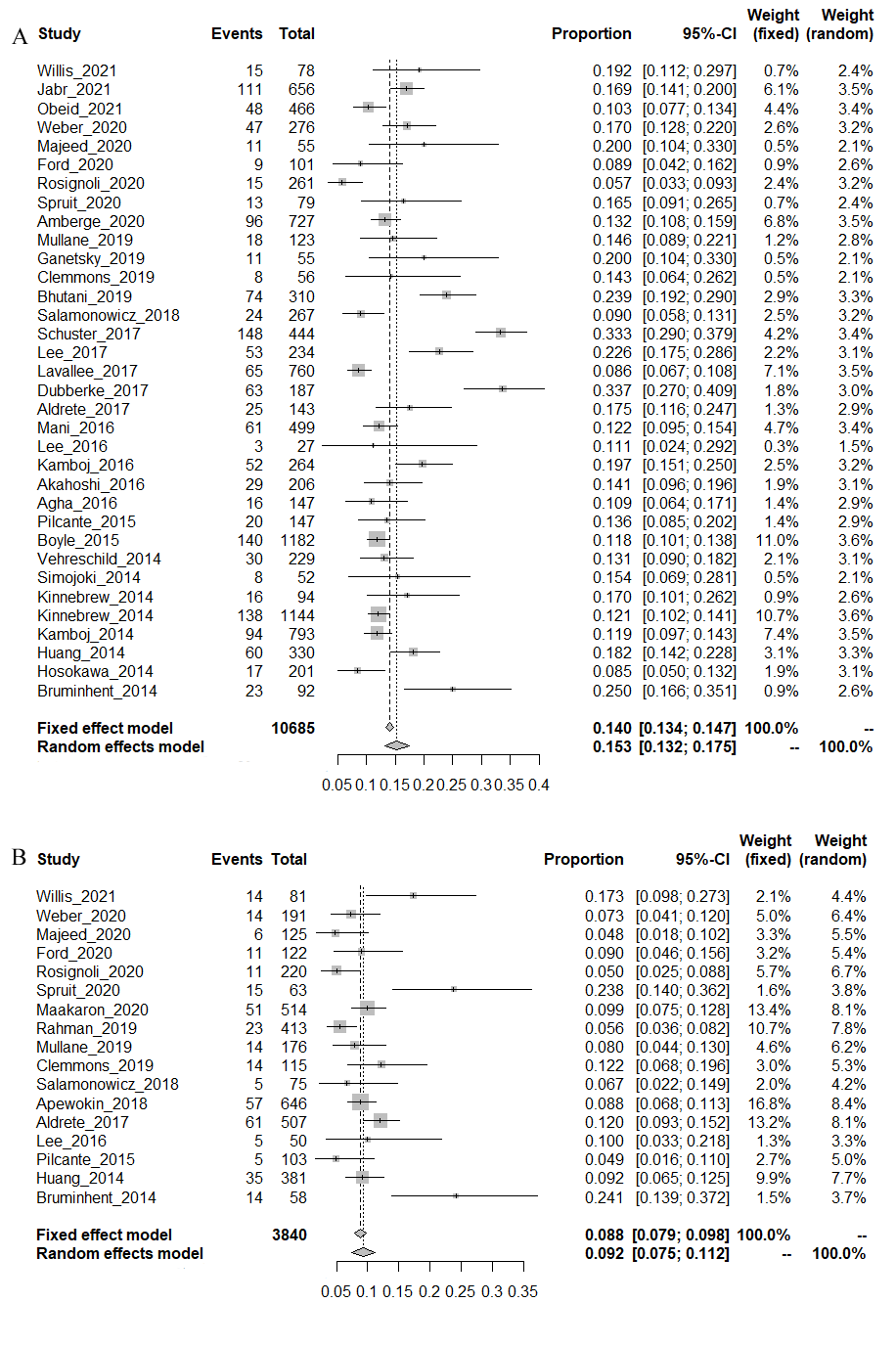

Supplement: Supplementary Figure 2 — Prevalence of CDI among allogeneic (A) and autologous (B) HSCT recipients. [file Image_2.tif]

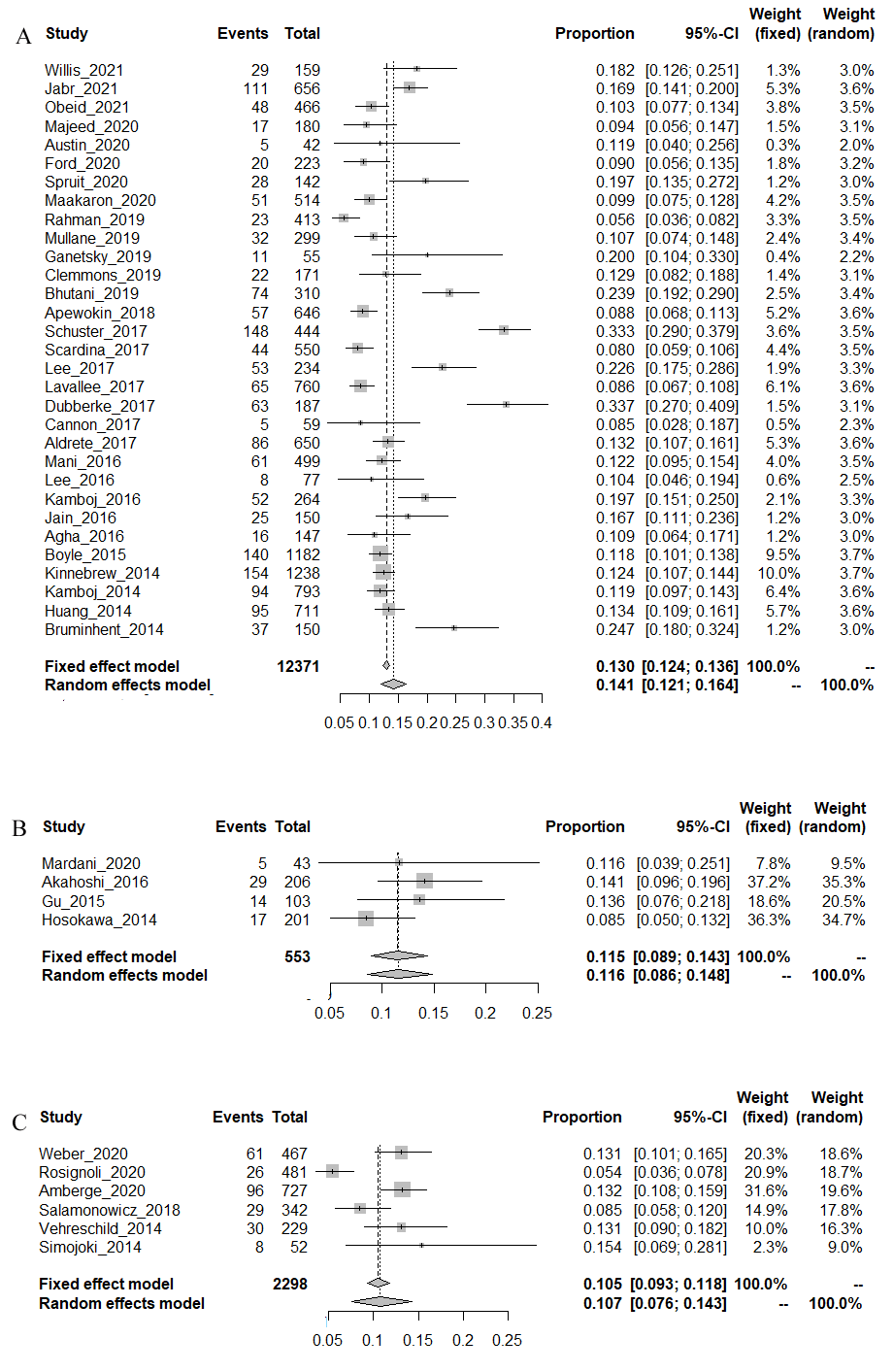

Supplement: Supplementary Figure 3 — Prevalence of CDI among studies in North America (A), Asia (B), and Europe (C). [file Image_3.tif]

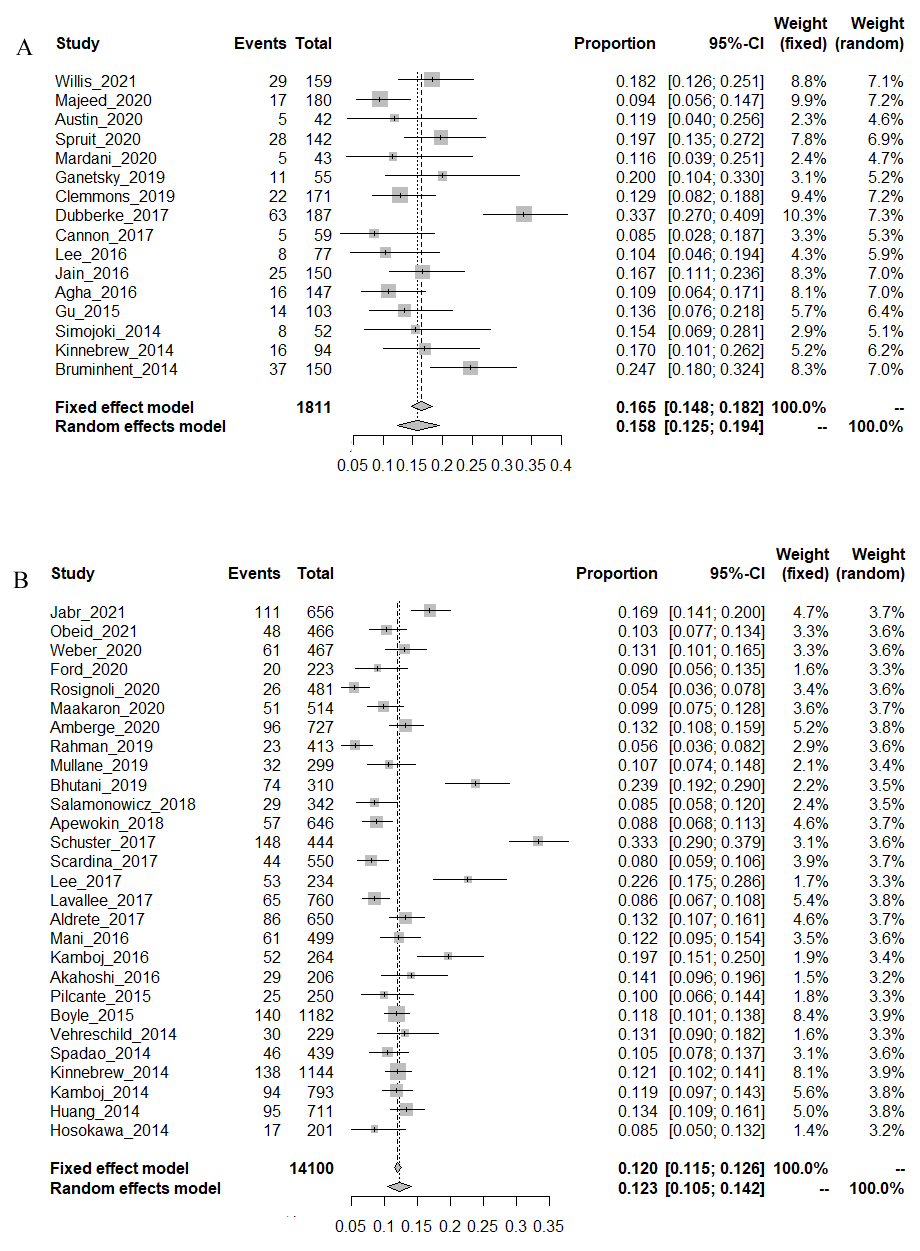

Supplement: Supplementary Figure 4 — Prevalence of CDI among studies with < 200 patients (A) and studies with ≥ 200 patients (B). [file Image_4.tif]

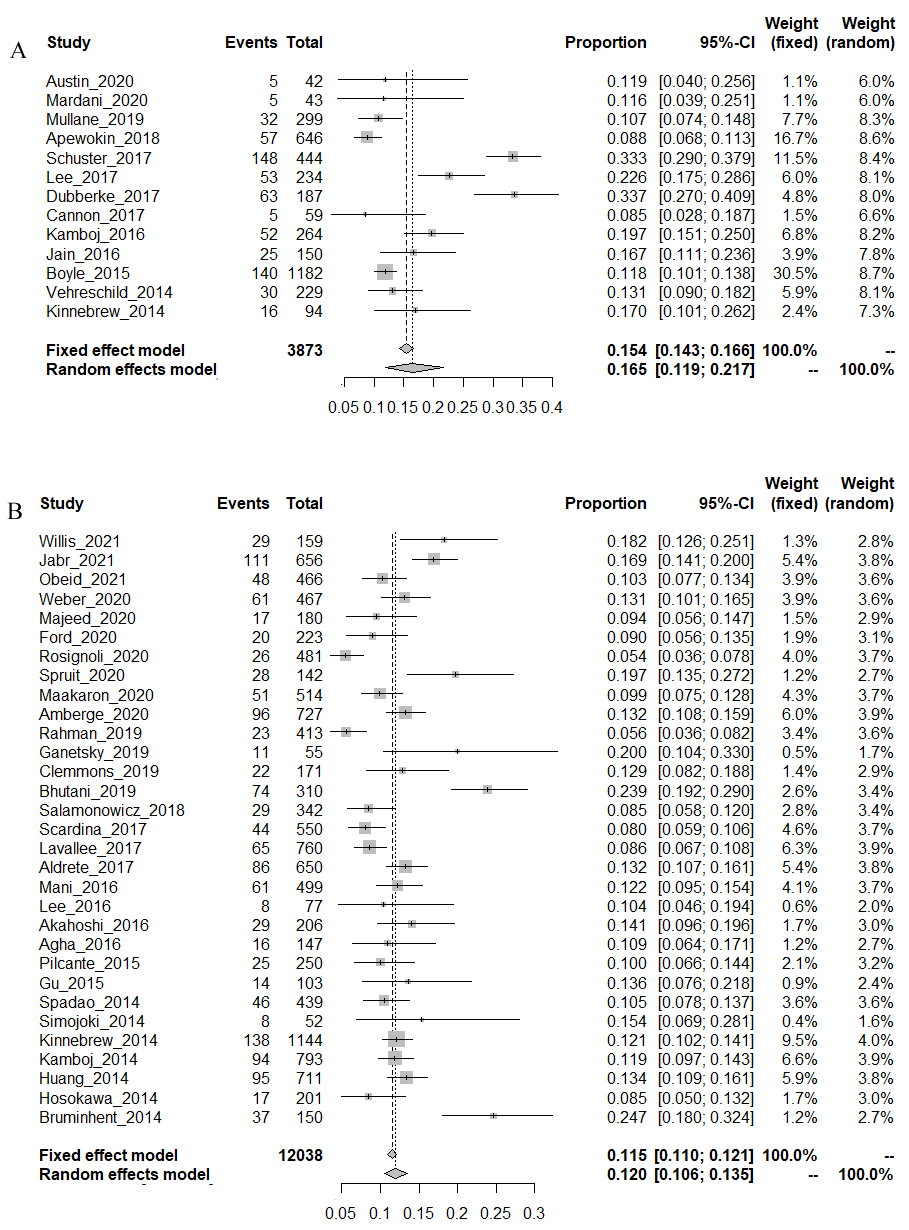

Supplement: Supplementary Figure 5 — Prevalence of CDI in prospective studies (A) and retrospective studies (B). [file Image_5.tif]

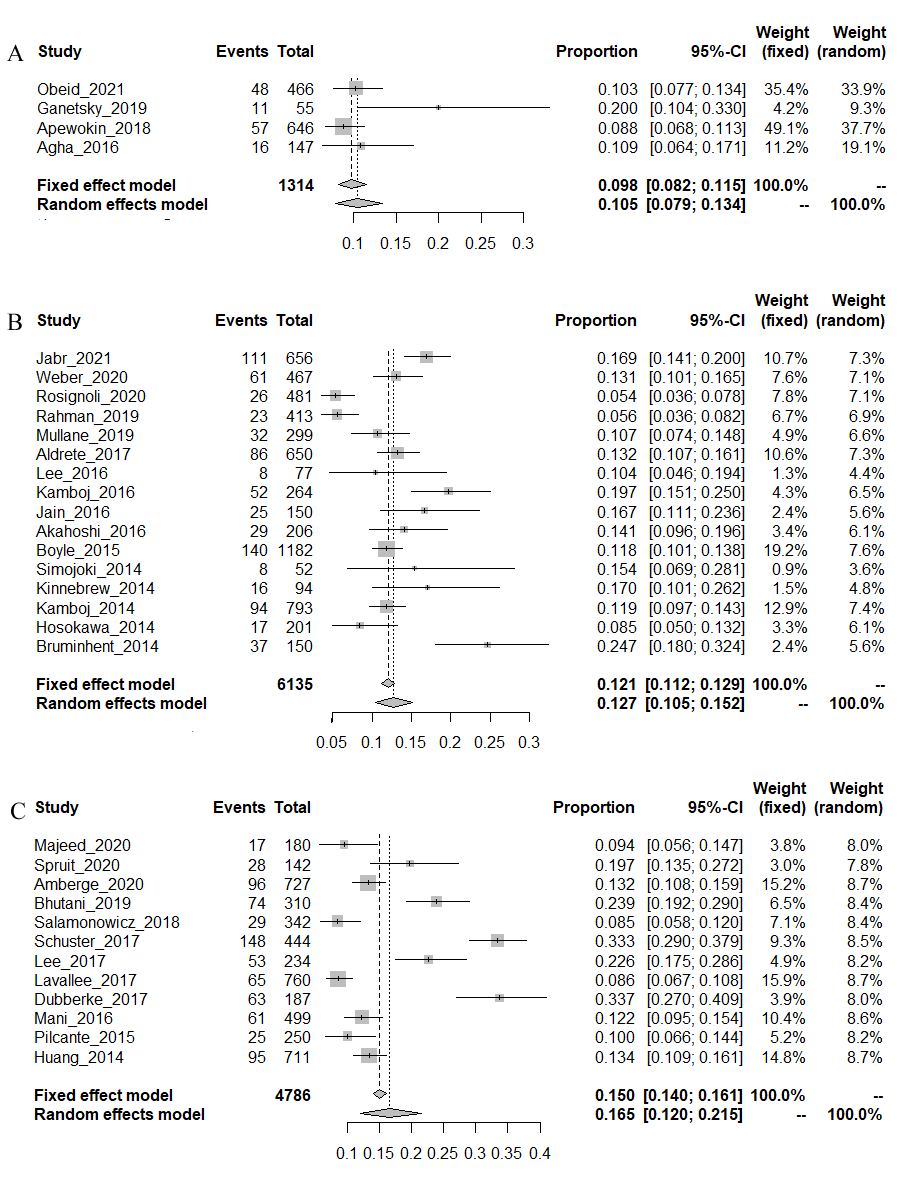

Supplement: Supplementary Figure 6 — Prevalence of CDI surveyed in the Early term (A), Middle term (B), and Long term (C). [file Image_6.tif]

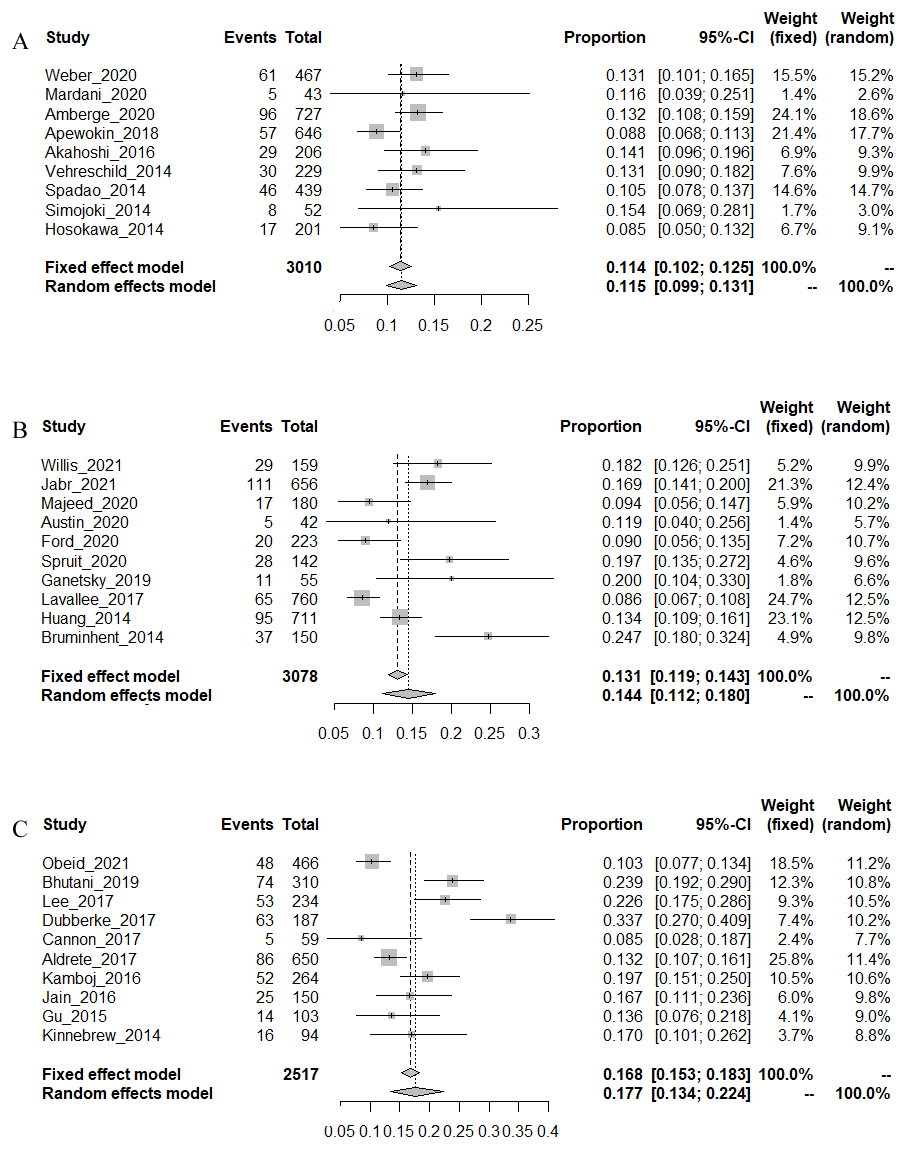

Supplement: Supplementary Figure 7 — Prevalence of CDI in studies with EIA used only (A), EIA + PCR/CC (B), and PCR used only (C). [file Image_7.tif]

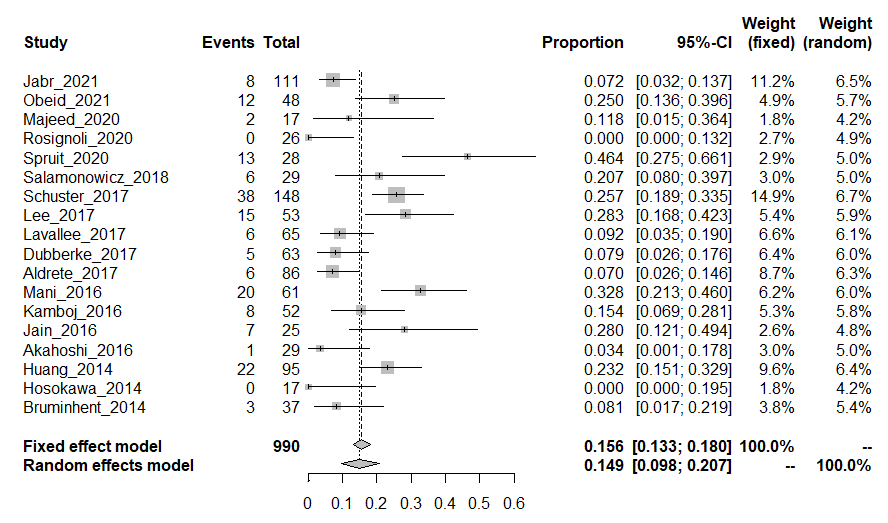

Supplement: Supplementary Figure 8 — Prevalence of recurrent CDI in studies. [file Image_8.tif]

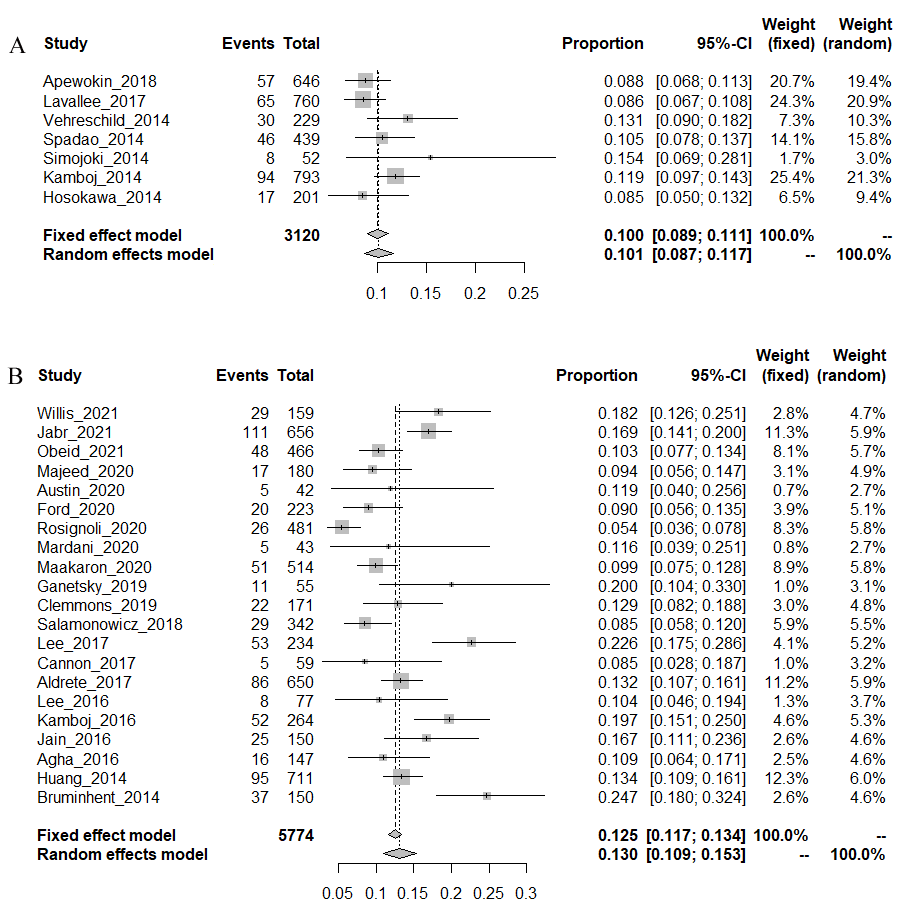

Supplement: Supplementary Figure 9 — Prevalence analysis of CDI in 1998-2010 (A) and 2010-2020 (B) year. [file Image_9.tif]
